# Prevalence of Anemia Among Adolescent Girls Residing in Rural Haryana: A Community-Based Cross-Sectional Study

**DOI:** 10.7759/cureus.21091

**Published:** 2022-01-10

**Authors:** Muthathal Subramanian, Sumit Malhotra, Shashi Kant, Kiran Goswami, Vanamail Perumal, Gaurishanker Kaloiya

**Affiliations:** 1 Community Medicine, Panimalar Medical College Hospital and Research Institute, Chennai, IND; 2 Centre for Community Medicine, All India Institute of Medical Sciences, New Delhi, IND; 3 Statistics & Demography, Obstetrics & Gynaecology, All India Institute of Medical Sciences, New Delhi, IND; 4 Clinical Psychology, All India Institute of Medical Sciences, New Delhi, IND

**Keywords:** prevalence, rural, menarche, adolescent girls, anemia

## Abstract

Background

Anemia continues to be a major public health problem in India despite multiple initiatives to address it among various vulnerable groups including adolescents.

Aim

This study was conducted to assess the prevalence of anemia among rural adolescent girls who had attained menarche.

Methods

The community-based cross-sectional study was conducted in 28 villages of Ballabgarh Block of district Faridabad, Haryana. From the computerized Health Management Information System data (HMIS), a random list of 363 adolescent girls was generated. Adolescent girls who had attained menarche were included in the study. Hemoglobin level was measured for all the consented or assented participants using a digital hemoglobinometer (HemoCue201+ photometer, HemoCue AB, Angelholm, Sweden).

Results

A total of 272 participants were enrolled in the study. Mean (SD) age at menarche was 13.2 (1.2) years. Prevalence of anemia among adolescent girls who had attained menarche was observed to be 71.7% (95% CI: 66.3 - 77.1) as per the WHO classification. Among the 195 anemic adolescent girls, severe, moderate, and mild anemia was observed in 4.8%, 41.2%, and 25.7%, respectively. In multivariable analysis, after adjusting for the age, the mother’s education was significantly associated with anemia (Adjusted Odds Ratio = 0.46, 95% CI: 0.22 - 0.96, p-value = 0.04).

Conclusion

The prevalence of anemia among rural adolescent girls who had attained menarche was high. Mother’s education status had a protective effect on anemia among adolescent girls.

## Introduction

There are 253 million adolescents in India, and 72% of them reside in rural areas [1]. Anemia is a serious public health challenge in India, even among adolescents [2]. According to National Family Health Survey-4 (2015-2016), the prevalence of anemia, i.e. hemoglobin level < 12gm/dL among adolescent girls (15-19 years), was 54% [3].

The adolescence period especially in girls is vulnerable to anemia due to the increased demand during the period of a growth spurt, inadequate dietary intake, vulnerability for helminthic infestation, and increased loss of iron during menstruation. Adolescent girls have a median iron loss of 12.5-15 mg per month during menstruation [4].

The nutritional concerns of adolescent girls, including anemia, are addressed by several flagship schemes and programs of the Government of India. These include the Integrated Child Development Services (ICDS) scheme, the Rajiv Gandhi Scheme for Empowerment of Adolescent Girls (RGSEAG)-SABLA, and more recently, POSHAN Abhiyaan and Anemia Mukt Bharat [5-7]. Since there are multiple programs addressing anemia, many specifically focusing on adolescent girls, there is a need for monitoring the prevalence of anemia for assessing the impact of such measures. Adolescent girls living in rural areas, especially those who have attained menarche, constitute the most vulnerable subgroup.

The Comprehensive National Nutrition Survey (CNNS, 2017) in the state of Haryana estimated anemia using venous samples in adolescent girls as 41%, almost twice than prevalence seen in boys (22%) [8]. Anemia causes easy fatigability, menstrual irregularities, and poor physical as well as mental health outcomes. Among adolescents in addition to this, there is a high chance of poor literacy outcomes. There is a paucity of literature about anemia among girls who had attained menarche. Against this background, this study aimed to estimate the prevalence of anemia and factors associated with it among adolescent girls (10-19 years) who had attained menarche and lived in rural areas of Ballabgarh Block of district Faridabad, Haryana.

## Materials and methods

A community-based cross-sectional study was conducted in 28 villages of Ballabgarh Block of district Faridabad, Haryana from November to December 2017. The total population of these villages in (2016-17) was 99,747. Data were obtained from the well-established database, i.e., Health Management Information System (HMIS). A computerized HMIS contained health and demographic data of all residents of these 28 villages. A more detailed description of the study site is available elsewhere [9]. The inclusion criterion was adolescent girls (10-19 years), who had attained menarche. Adolescents that were pregnant, unable to comprehend, or not contactable after three home visits were excluded. Assuming the prevalence of anemia (p) among adolescent girls as 87% [10], relative precision of 5%, 95% confidence level, and 15% non-response rate, the minimum required sample size was 242. The menarcheal status among adolescents was unknown. Hence, the sample size was further inflated by 50%. The final required sample size thus obtained was 363. There were 7,748 adolescent girls in the HMIS database. A list of total adolescent girls (N = 7,748) was generated from the HMIS database of the rural field practice area, Ballabgarh Block. Using this sampling frame, a simple random sample of 363 adolescent girls was drawn in the Microsoft (MS) Excel 2016 (Microsoft® Corp., Redmond, WA) using =RAND () function, meaning random. This function was used to generate a random list of adolescent girls from the sampling frame. Once the random list was made, girls were sorted village-wise and were approached within the community with the help of a local village-level health worker for the interview.

Data collection

The study was conducted for two months by the trained investigator [MS]. A self-developed, pre-tested, semi-structured questionnaire that contained items relevant to study objectives viz. demographic details (including socioeconomic status), menstrual history and intake of prophylactic iron and folic acid supplements, and deworming was used. The study instrument was not validated separately, though was pre-tested and piloted in the adjacent study area before use. Hemoglobin level was measured by a digital hemoglobinometer by the study investigator [MS]. Training and supervisory support for the execution of the data collection work was provided by senior investigators within the team [SM, SK, KG].

Hemoglobin estimation (gm/dl) was done by using the digital hemoglobinometer method, i.e., Hemocue201+ (Hemocue AB, Angelhom, Sweden). This is a point of care diagnostic method utilized in field settings that are operated through the battery. It works on the principle of modified azide methemoglobin determination using a specially designed micro cuvette with dried reagents. The hemoglobin method is estimated using absorbance levels at two wavelengths of 570 nm and 880 nm for turbidity compensation in the blood sample [11]. Using a sterilized gauge piece and lancet, the initial two drops of blood were discarded, and the third drop of blood was placed in the HemoCue slide by the investigator (MS), and then in the digital hemoglobinometer. It displayed hemoglobin levels immediately which was noted in the log sheet of the study participant and informed to the participant.

Anemic adolescents were prescribed tablet IFA (Iron Folic Acid). Those requiring further management were referred to the nearest government health facility. The socio-economic status was assessed using the Uday Pareek Scale updated for the year 2017 [12]. Participants in the random list were paid a home visit and screened for eligibility criteria. The participants and available parents/guardians were explained about the study objective and procedure. The assent was taken from the participants if they were younger than 18 years. Written informed consent was taken from the study participants aged 18 years or older and the participant’s parents/guardians if the age of the participant was less than 18 years.

The severity of anemia was classified as per the World Health Organization (WHO) recommendation. In brief, anemia was classified as mild if the hemoglobin level was 11.0-11.4 gm/dL for those aged 10 to 11 years, and 11.0-11.9 gm/dL for those aged 12-19 years. Anemia was classified as moderate when the hemoglobin level was 8.0-10.9 gm/dL, and severe if the hemoglobin level was <8 gm/dL [2].

Institute Ethics Committee (IEC) approved the study protocol (Reference number: IECPG-224/23.08.2017, RT-07/07.09.2017).

Statistical methods

Data were entered in Microsoft Excel 2010 and cleaned data were analyzed using STATA version 12.0 statistical software (StataCorp LLC, College Station, TX). Continuous variables were tested for normality assumption using the Kolmogorov-Smirnov test. For normally distributed data, descriptive measures such as mean, standard deviation (SD), and range values were calculated. For non-normally distributed data, median and inter-quartile range (IQR) were calculated. Median values were compared using the Kruskal-Wallis test or Wilcoxon signed rank-sum test as appropriate. For categorical data, associations were ascertained using Chi-square/Fisher’s Exact test as appropriate and odds ratio with 95% confidence interval (CI) limits were calculated. Bivariable analysis was performed to find an association between anemia and a few selected variables like age, education, mother’s education, and socioeconomic status. Significant variables (p-value ≤ 0.2) were entered in the multivariable logistic regression model to calculate the adjusted odds ratio and 95% CI. A p-value < 0.05 was considered statistically significant.

## Results

There were 274 eligible participants out of a total of 363 participants. Two eligible participants refused to give consent. Finally, 272 participants were included in the study. The majority of them (67%) were in the late adolescence group (15-19 years). The mean (SD) age of the participants was 15.5 (±1.8) years. The majority (88.2%) of the participants were students, and 44.9% were attending middle school. A total of 2.2% of adolescent girls were married. A total of 43% of mothers of the participants were illiterate, 21.7% had completed primary schooling, and 2.6% of them were graduates. According to the Udai Pareek Scale [12], the majority (48.2%) of the participants belonged to the middle class and 38.2% to the lower middle class. Table 1 shows the selected demographic details of the participants.

**Table 1:** Distribution of the study participants by socio-demographic characteristics (N = 272)

Variable	Category	Number (%)
Age group	Early adolescence (10-14 years)	90 (33.0)
	Late adolescence (15-19 years)	182 (67.0)
Education (completed school years)	Illiterate	1 (0.0)
	Primary	6 (2.0)
	Middle school	122 (45.0)
	High school	89 (33.0)
	Secondary school	54 (20.0)
Occupation of the participant	Student	240 (88.0)
	Others*	32 (12.0)
Marital status	Unmarried	266 (98.0)
	Married	6 (2.0)
Religion	Hindu	266 (98.0)
	Muslim	4 (1.0)
	Sikh	2 (1.0)
Education of the participant’s mother	Illiterate	117 (43.0)
	Primary	58 (21.0)
	Middle school	40 (15.0)
	High school	29 (11.0)
	Secondary school	21 (8.0)
	Graduate	7 (3.0)
Socio-economic status according to Udai Pareek Scale	Upper middle (33-42)	34 (13.0)
	Middle (24-32)	131 (48.0)
	Lower middle (13-23)	104 (38.0)
	Lower (<13)	3 (1.0)

The mean (SD) hemoglobin level of the participants was 10.9 (±1.64) gm/dL. The prevalence of anemia among adolescent girls who had attained menarche was 71.7% (95% CI: 66.3 - 77.1) as per the WHO classification. Among the 195 anemic participants, 13 (6.7%), 112 (57.4%) and 70 (35.9%) had severe, moderate and mild anemia, respectively. The difference between the severity of anemia and age groups was statistically not significant (p-value = 0.9) (Table 2).

**Table 2:** Distribution of category of anemia by age group (N = 272) *p-value = 0.9 using Chi-square test, Chi-square value - 0.38, degree of freedom (df) = 3

Age group in years*	Normal	Mild	Moderate	Severe	Total
10-14 n (%)	27 (30.0)	24 (26.7)	35 (38.9)	4 (4.4)	90 (100)
≥ 15 n (%)	50 (27.5)	46 (25.3)	77 (42.3)	9 (5.0)	182 (100)
n (%) (95% Confidence Interval)	77 (28.3) (22.7 – 33.3)	70 (25.7) (19.9 – 30.1)	112 (41.2) (35.2 – 46.8)	13 (4.8) (2.3 – 7.3)	272 (100)

Among the school-going participants, 78 of them (29.9%) received and consumed iron-folic acid (IFA) supplements. The reason for not consuming was side effects (6.1%), and for the rest of the participants studied in the private schools. Deworming was done periodically among 26.4% of participants. Self-reported symptoms such as premenstrual syndrome (61%), and health problems during menstruation (73%) were found among the study participants. Less than 1/6th (13.3%) of the participants had an irregular menstrual history in terms of regularity of the menstrual cycle and menstrual blood flow.

The prevalence of anemia was more than 50% among late adolescents. The prevalence of anemia was not associated with age category. The participants who were educated above middle school, belonged to lower socio-economic status, and whose mothers were illiterate had a higher prevalence of anemia in each anemia category. However, the difference was statistically not significant (Table 3).

**Table 3:** Distribution of anemia by selected socio-demographic variables * p-value ≤ 0.20, unadjusted odds ratio for reference category used for comparison is marked as 1.00

Variable	Category (n)	Anemia present (n = 195) n (%)	Unadjusted odds ratio	95% CI	p-value
Age	Early adolescence (90)	63 (32.0)	1.00	-	-
	Late adolescence (182)	132 (68.0)	1.13	0.64 – 1.97	0.66
Education of the participant	≤ 8^th^ class (129)	89 (46.0)	1.00	-	-
	≥ 9^th^ class (143)	106 (54.0)	1.28	0.75 – 2.18	0.34
Education of the mother	Illiterate (117)	90 (46.0)	1.00	-	-
	1-8^th^ class (98)	69 (35.0)	0.82	0.44 – 1.51	0.53
	9^th^ and above (57)	36 (18.0)	0.55	0.27 – 1.09	0.08*
Socio-economic status	Middle and upper middle (165)	114 (58.0)	1.00	-	-
	Lower and lower middle (107)	81 (42.0)	1.39	0.80 – 2.41	0.23

Bivariable analysis between anemia and selected socio-demographic variables revealed that the education of the participant’s mother was associated with anemia (p-value ≤ 0.20) (Table 3). Logistic regression analysis between the education of the participant’s mother and anemia after adjusting for the participant’s age was performed. The odds of having anemia among the participants born to educated mothers was 54% less compared to participants born to illiterate mothers (Table 4).

**Table 4:** Logistic regression between anemia and education of the mother *p-value <0.05 after adjusting for participant’s age in completed years, adjusted odds ratio for reference category used for comparison is marked as 1.00

Variable	Category (n)	Anemia present (n = 195) n (%)	Adjusted odds ratio	95% CI	p-value
Education of the mother	Illiterate (117)	90 (46.0)	1.00	-	-
	1-8^th^ class (98)	69 (35.0)	0.78	0.42 – 1.46	0.44
	9^th^ and above (57)	36 (18.0)	0.46	0.22 – 0.96	0.04*

There was no significant association between IFA intakes through Weekly Iron and Folic Acid Supplementation (WIFS) in school, deworming status, age at menarche, menstrual history, menstrual disorders viz. irregular cycles, hypomenorrhea and anemia.

## Discussion

This study was conducted to assess the prevalence of anemia among adolescent girls who had attained menarche and were living in a rural area of Haryana. The hemoglobin levels as prescribed by the WHO were used to diagnose anemia using a digital hemoglobinometer in this study. For girls aged 10-11 years, hemoglobin level <11.5 gm/dL, and for girls aged 12 years and above, hemoglobin level <12.0 gm/dL was used for diagnosing anemia [2]. The prevalence of anemia was 71.7% (95% CI: 66.3 - 77.1). A community-based study in 2017 conducted among rural adolescent girls (12-19 years) reported a high prevalence of anemia (88.3%). The study population in this study was school girls from Haryana [13]. A higher prevalence similar to this study was reported from other states also such as by Ahankari et al. [10] in Maharashtra (87%), Rati and Jawadagi [14] in Karnataka (80%), Koushik et al. [15] in Andhra Pradesh (77.3%), and Gupta et al. [16] in Chhattisgarh (76.3%). The higher prevalence of anemia in the present study could be due to the inclusion of late adolescents as the study population. Late adolescents experience physical and biological changes following puberty that increase the demand for nutrients. Failure to provide the increased demand for iron could lead to anemia among late adolescents.

The reported range of anemia among adolescent girls varies with the study settings, i.e., community-based, school-based, or hospital-based. Among community-based rural Indian studies, the reported prevalence of anemia varies from 21% to 90% [10, 17-19]. The reported prevalence of anemia is still higher (44% to 100%) among school-based studies in India [13,14,16,20,21]. Two studies from the health care facility reported the prevalence of 48.6% [22] and 77% [15] among adolescent girls from southern India. Thus, irrespective of the study setting, the prevalence of anemia among adolescent girls in India is unacceptably high.

A school-based study that had used the same tool as in the current study for estimation of hemoglobin level, reported the prevalence of anemia as 44%. The lower prevalence observed in that study could be due to the exclusion of adolescents aged 15 years or older [20].

The prevalence of severe anemia in the present study was 4.8%. This study's findings were unlike other studies that have reported a much higher figure (7.1% to 13%) [13-15, 18, 21, 22]. The precise reason for such a difference is not obvious. However, part of the differences could be explained by the differences in the study setting, and tool used to measure hemoglobin levels. Hemocue201+ has high sensitivity (93%), acceptable specificity (76%), and moderate concordance with the gold standard Sysmex autoanalyzer for measuring hemoglobin [23].

The mother’s educational status had a protective effect on anemia in an adolescent girl. Other studies have also reported similar findings [16, 24]. The beneficial effect of a mother’s education has been reported on many other health parameters, including infant and child mortality. A mother’s education may be reflective of an overall higher level of socio-economic status that in itself has a protective effect against anemia. The impact of socio-economic status on anemia among adolescent girls is well established [14, 16, 21, 22, 24].

There was no association between anemia and iron-folic acid intake, deworming status, menarcheal age, menstrual history, and menstrual disorders. This finding was contrary to the findings reported by other studies. Studies have reported that menstrual factors such as usage of more pads per day (>2 pads), increased flow per cycle (≥5 days) had a significant association with anemia among adolescent girls [14, 20, 24].

Limitations

The findings of this study may not be extrapolatable to urban areas and also, they can be generalized only to the adolescent girls who attained menarche within rural communities of Haryana. The self-reported history of deworming and iron-folic acid intake, and menstrual problems were taken, which had the possibility of recall bias. We did not ascertain the etiology of anemia, i.e., microscopic typing, and serum indicators that may be needed for determining the burden of different types of anemia in this area. The sample size was calculated for the primary objective that was related to the prevalence of anemia, hence it might be inadequate to establish the association between selected variables and anemia. Future comprehensive studies will be needed to gauze the magnitude from multiple settings and geographic regions along with biochemical analysis to present the burden of specific types of anemia. Our study had a few strengths also. Simple random selection from a known sampling frame and negligible refusal rate ensured that the findings were extrapolatable to the area from which the samples were drawn. A digital hemoglobinometer was used whose sensitivity and specificity in field conditions are acceptable. Hence, the prevalence rate seems to be valid.

## Conclusions

The prevalence of anemia among rural adolescent girls who had attained menarche was high. Mother’s education status had a protective effect on anemia among adolescent girls. Involvement of mothers in educating about corrective measures for anemia reduction should be promoted. The etiology of anemia among these rural adolescent girls who had attained menarche needs to be investigated and appropriate preventive and therapeutic measures may be instituted.

## Appendices

**Table 5:** Study Questionnaire/ Interview Proforma

PARTICIPANT DEMOGRAPHIC DETAILS:					
1	Age in completed years: If available, Date of birth:______		8	Family type: [1- single/2- joint or size up to 5 or any other distinctive features/3- joint] ____	
2	Participant ID/ Village name/ Household no: ___________/_____________/_____________		9	Caste: [1- SC/2- low caste/3- artisan caste/ 4- agriculture caste/5- prestige caste/6- dominant caste] ___	
3	Marital status: [0-unmarried/1-married] ______		10	Land: [0- no land/1- <1 acre/2- 1-5 acre/3- 5-10 acre/4- 10-15 acre/5- 15-20 acre/6- 20 & > acre]______	
4	Education of the participant: (completed schooling) [0- illiterate/1- 1^st^ to 5^th^ /2- 6^th^ to 8^th^ /3- 9^th^ to 10^th^ /4 – 11^th^ to 12^th^ / 5- graduate] _____		11	Social participation: [0- none/1- member of 1 organization /2- member of > 1 organization / 3- office holder in such an organization/4- wide public leader] _____	
5	Occupation of the participant: [0- student/1- housemaker/2- labor/3- others (specify)] ___________		12	House: [0- no house/1- hut/2- kutcha/3- mixed/ 4- pukka/5- mansion] _______	
6	Education of the mother/ guardian of the participant: [0-illiterate/1-1^st^ to 5^th^ /2-6^th^ to 8^th^ /3- 9^th^ to 10^th^ / 4 – 11^th^ to 12^th^ / 5-graduate] __________		13	Farm power:[1- no draught animals/2- 1-2 draught animals/4- 3-4 draught animals/6- 5-6 draught animals] _______	
7	Religion: [0-Hindu/1-Christian/2-Muslim/3-sikh /4-others(specify)] _______		14	Material possessions: [0-bullock cart/1-cycle/2-radio/3-chairs/4-mobile phone/5-television/ 6-refrigerators] ______	
DETAILS OF MENSTRUAL HISTORY:					
15		Have you attained menarche? [1-yes.0-no] If yes, go to the next question.			
16		Age at menarche in completed years			
17		What is your average duration of menstruation in days per menstrual cycle?			
18		In the last period, how many days was the menstrual flow?			
19		What is the interval in days between two cycles?			
20		What are the health-related problems you faced during menstruation?			Dysmenorrhea /Menorrhagia / Hypo menorrhea/Oligomenorrhea/ Polymenorrhea/ Premenstrual symptoms/ Backache /Others (specify)______________
MEDICATION HISTORY RELATED TO WIFS:					
21		History of deworming in the last 6 months [1-yes.0-no]			
22 a		Have you taken iron-folic acid (IFA) through WIFS in school? [1-yes.0-no]			
22 b		If yes, the number of tablets consumed in the last 3 months			
23		Hemoglobin value in gm/dL			
